# The Crucifixion of Love

**Published:** 1918-04-06

**Authors:** 


### The Hospital

The Workers' Newspaper of Administrative Medicine and Institutional Life, Administration, National Insurance and Health.

No. 1661, Vol. LXIV. SATURDAY, APRIL 6, 1918. PRICE ONE PENNY

### THE CRUCIFIXION OF LOVE.

The Bishop of London preached at St. Paul's, Knightsbridge, on Good Friday, having just returned from three days spent with the boys at Harrow School, who had poured out their doubts and difficulties to their Bishop, and had asked where God was and whether He was to blame for the war. The Bishop said: Many people had that doubt. God was only responsible in the sense that He made a world in which war was possible. It was too much that His tremendous generosity should be turned against Himself, too much that we should take every little evidence of vile hate and bring that up against His infinite love. This is the Devil's war, not God's, but God himself is a victim of it. Thank God, what connects that unbroken line of our boys and men peering into the smoke of the morning, with Calvary? It is that in both you see the crucifixion of love. These men had gone to the rescue of womenkind, the rescue of the weak, the rescue of love. They had gone with undaunted hearts and fearless courage, because love was being crucified, in Europe, to-day.

The strain and doubt and awfulness of the great battle, proceeding as we write, have aroused the nation, and are awakening the whole civilised world to the wickedness of the present war, which originated with Germany and was forced on the world by Germany, for Germany's private ends and ambitions. The war correspondents have given in the last few days graphic descriptions of what it means to the British and Allied troops to face the German rushes, in which the Chiefs of the German Army, led by the Kaiser himself, have displayed throughout the most callous and complete disregard for their own men, whom they have treated as mere fodder for the guns of the enemy. This course is taken, in pursuance of their absorbing ambition to become masters of the world, and the slave-drivers of civilised man. The descriptions in question are thrilling, moving, awful. The present writer has been over the country where these battlefields are, and is intimately acquainted with what the conditions of these old charnel grounds were before this battle began. This circumstance aids the realisation that, in addition to the fighting, the condition of the terrain, in this wilderness of desolation, created by the Germans in their previous occupation of it, and the fighting which resulted, meant to our troops and the French an aggregation of horrors and difficulties which are well-nigh indescribable, and which no one at home, however fertile their imagination, can realise or understand. The actual condition of most of the ground, where the fighting has been, is such that, by the designed and laboured action of the Germans, one of the finest agricultural districts in France has been so destroyed, defiled, and corrupted, as to make it impossible, the Germans hope, that it can be used for residential or agricultural purposes for at least one hundred years.

We think it well when the mind and attention of the whole nation and the peoples of all classes throughout the Empire and the United States of America, and even neutral countries, is concentrated upon the battle, that these facts should be recalled to-day. That everyone may have an opportunity to realise the heroism and wonderful grit and determination displayed by the British and French soldiers, and the American soldiers, too, who have taken part in the fight, resting, as these high qualities undoubtedly do, upon the sense in all our warriors, that the Bishop's words are, in fact, true. For the Crucifixion of Love, in Europe, to-day, is a demonstrable fact, which it is their proud privilege to resist to the death, regardless of their lives and of everything save the call upon them to stand for God, in this great battle. Our men, the French soldiers, the Americans who are fighting, feel and realise the glory of being privileged to engage in this battle, believing, as they properly do, that they are fighting to retain for their countries, for their fellow-citizens, and for all they love best, the right to be free. To them, too, will be the glory of giving, all that they have preserved to them untarnished, to the principle of love, for which Christ died, and without which the world would cease to be possible, for every member of the race who believes in God, righteousness, a clean life here, and a future beyond the grave.

Many of our readers, from their experience and knowledge, cannot fail to realise the charnel condition of the ground on which the greatest of battles is being fought. They will apprehend what the actual condition of that ground must necessarily become, as a result of the awful slaughter of human beings, of horses, and other things. They will be able to follow the results of the daily and ever-increasing slaughter and horrors, attendant on the murderous system, which the Germans have mercilessly pursued throughout. In view of this knowledge, they may well be considering what is a possible, and even probable, result which may speedily arise in the hygienic sense. It was strongly urged that Europe originally owed the epidemic of influenza, to which its people have been subjected during the last twenty-five years or so, to the death of some two million Chinamen from an epidemic which occurred in that country a few months before influenza reached Europe. Is it possible to hope that, in the conditions of the fighting, and the awful slaughter of hundreds of thousands which has been and is taking place, an epidemic can be prevented, even assuming that the Germans were in a position to enforce, with their credited thoroughness, all the precautions against it known to science? We think these considerations are well worthy of attention. They are no doubt in the minds of our own health authorities at the Front. The outlook at the moment is necessarily so grave that, the question arises, whether an outbreak of disease may not have a material influence upon the fighting men of the enemy, especially, for they will occupy the territory where the results of such an outbreak, if it occurs, may undoubtedly prove to be devastating and conclusive.

